# Intensifying the response of distributed optical fibre sensors using 2D and 3D image restoration

**DOI:** 10.1038/ncomms10870

**Published:** 2016-03-01

**Authors:** Marcelo A. Soto, Jaime A. Ramírez, Luc Thévenaz

**Affiliations:** 1 EPFL Swiss Federal Institute of Technology, Group for Fibre Optics,, SCI-STI-LT, Station 11, CH-1015 Lausanne, Switzerland

## Abstract

Distributed optical fibre sensors possess the unique capability of measuring the spatial and temporal map of environmental quantities that can be of great interest for several field applications. Although existing methods for performance enhancement have enabled important progresses in the field, they do not take full advantage of all information present in the measured data, still giving room for substantial improvement over the state-of-the-art. Here we propose and experimentally demonstrate an approach for performance enhancement that exploits the high level of similitude and redundancy contained on the multidimensional information measured by distributed fibre sensors. Exploiting conventional image and video processing, an unprecedented boost in signal-to-noise ratio and measurement contrast is experimentally demonstrated. The method can be applied to any white-noise-limited distributed fibre sensor and can remarkably provide a 100-fold improvement in the sensor performance with no hardware modification.

## Abstract (web summary)

Optical fibres offer a way of sensing across a wide area, providing a spatial and temporal map of the measured parameter. Here, the authors use image and video processing to remove noise and increase the contrast in such a distributed optical fibre sensor, improving the signal-to-noise ratio by a factor of 100.

## Graphical abstract

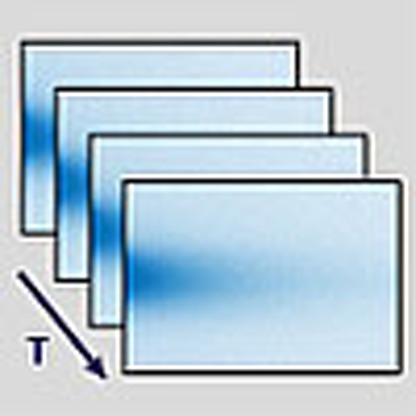

Distributed fibre sensors^1^,^2^ exploit specific optical effects activated along optical fibres, to obtain a spatially distributed profile of environmental quantities such as temperature, strain, pressure, electromagnetic fields and so on. This feature offers unique attributes and capabilities compared with conventional discrete sensing methods^1^,^2^. Conventional distributed fibre sensors can be classified in a wide range of types^2^ based on the nature of the exploited optical effect. There exist types based on optical absorption, fluorescence, evanescent field or interferometers, among others^1^,^2^; however, most of the distributed sensors use natural scattering processes^3^,^4^ present in optical fibres along with interrogating methods based on time-domain^5^ or frequency-domain^6^ reflectometry. These are sensors essentially based on Rayleigh scattering^7^,^8^,^9^,^10^,^11^, spontaneous Raman scattering^12^,^13^,^14^ and spontaneous or stimulated Brillouin scattering^15^,^16^,^17^,^18^,^19^,^20^,^21^,^22^,^23^,^24^. The spatial resolution of distributed sensors is primarily determined by the bandwidth of the interrogating signal, which is typically traded off with the power contrast in the detected signal. Under an optimized configuration, the best performance attained by any distributed sensor is ultimately determined by the signal-to-noise ratio (SNR) of the measurements^16^,^24^. Therefore, to ensure a given measurement quality (defined by the measurand resolution), a minimum SNR has to be secured in the system^24^,^25^. This imposes an important tradeoff between spatial resolution and sensing range, which affects all kinds of distributed fibre sensors^1^,^2^,^25^,^26^,^27^. Sensors with high (that is, sharp) spatial resolution are inherently limited by the short interaction time and low energy involved in the scattering process (locally activated at each fibre position), enabling measurements only along short ranges^19^,^20^ (typically below 1 km). On the other hand, sensors with metric resolution can typically operate over many tens of kilometres of optical fibre. In this case, the SNR and quality of the measurements are basically limited by the onset of nonlinear effects^28^,^29^,^30^,^31^,^32^, which constraint the maximum optical power launched into the sensing fibre, and by the fibre attenuation, which leads to an exponentially decaying sensor response and poor measurement contrast at the end of long optical fibres^24^,^25^.

The clear understanding reached in recent years on the factors ultimately limiting the sensor capabilities has motivated researchers to propose and demonstrate specially designed methods for performance enhancement^33^,^34^,^35^,^36^,^37^,^38^,^39^,^40^,^41^,^42^,^43^,^44^,^45^,^46^,^47^,^48^,^49^,^50^,^51^,^52^,^53^,^54^. Among several advanced techniques, methods such as distributed Raman amplification^34^,^35^,^36^,^37^, optical pulse coding^38^,^39^,^40^,^41^,^42^,^43^ or different signal processing methods^44^,^45^,^46^,^47^,^48^,^49^,^50^,^51^ have resulted in implementations outperforming classical standard configurations. Whereas each of these methods can individually provide up to about 10–12 dB SNR enhancement, a higher improvement can only be reached by a proper combination of several of those techniques^52^,^53^,^54^, at the cost of complex and expensive implementations.

Among several existing methods, signal processing techniques, such as optical pulse coding^38^,^39^,^40^,^41^,^42^,^43^, wavelet transform^44^,^45^,^46^,^47^,^48^ and Fourier transform^49^ have demonstrated to be very efficient tools to remove noise. However, their exploitation for distributed sensing has been restricted so far only to one-dimensional (1D) arrays of data. Those techniques can be readily applied, for instance, to Raman-distributed fibre sensors^38^,^44^, owing to the 1D nature of the acquired data (corresponding to 1D traces of the anti-Stokes, Rayleigh and Stokes backscattered light^12^,^13^,^14^). In the case of Brillouin- and Rayleigh-based distributed sensors, in which time and frequency are scanned, signal processing has been used to denoise individual longitudinal traces (that is, at a fixed scanned frequency) independently from each other^39^,^40^,^41^,^45^, the measured local spectrum at each fibre location^47^ or the retrieved measurand profile^48^. Although methods such as time–frequency coding^42^,^43^ take advantage of the double scanning (fibre position and pump–probe frequency detuning) required in Brillouin sensing, the provided SNR enhancement is basically given by the ability of the code to reduce noise in a 1D array of data. Indeed, none of the existing methods for performance enhancement exploit the redundancies and correlations contained in the multidimensional domain of the measured information. This is so far a feature of distributed fibre sensors that has been completely unexplored in the state-of-the-art; however, as such measurements contain repeated structures of information in a multidimensional domain (time, frequency and position), they can be smartly and efficiently exploited to improve the SNR of the measurements.

In the following, we propose and experimentally demonstrate an approach that exploits correlated patterns of information and their high degree of redundancy for enhancing the measurement quality and performance of distributed optical fibre sensors. In particular, this approach makes use of image and video enhancement processing for removing noise and increasing the contrast of noisy measurements obtained by any kind of distributed sensor. To the best of our knowledge, this is the first time that such a multidimensional approach is used to restore information and enhance the capabilities of distributed fibre sensors. Here we demonstrate an unprecedented boost in SNR, which can reach two orders of magnitude (that is, ∼20 dB SNR enhancement), being equivalent or even superior to the use of extensively complex hardware sophistications but at a minor fraction of the cost. Any dB gained in SNR can be used to improve the sensor performance, that is, to extend the range, to sharpen the spatial resolution, to reduce the measurement time (reducing the number of averages), or simply to improve the measurand accuracy^24^. The technique can be applied to any conventional or advanced sensor in which the acquired data can be arranged in a two-dimensional (2D) or three-dimensional (3D) data structure. This includes any possible configuration for distributed fibre sensing based on, for example, faint long gratings^55^, Rayleigh^7^,^8^,^9^,^10^,^11^, Raman^12^,^13^,^14^ or Brillouin^15^,^16^,^17^,^18^,^19^,^20^,^21^,^22^,^23^,^24^ scattering (or any combination of them); however, the use of the method can also be extended to any reflectometry-based technique for fibre characterization^5^,^6^ as well as for quasi-distributed or multiplexed sensors, such as arrays of fibre Bragg gratings^56^, in which the measured information can be arranged in a 2D or 3D data structure.

## Results

### 2D image processing for sensor data restoration

**Figure 1:**
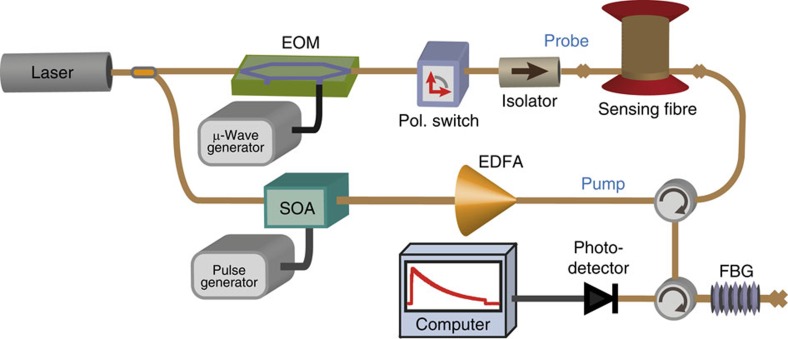
Experimental setup of a basic BOTDA sensor. The light of a conventional distributed-feedback laser operating at 1,550 nm is split into distinct branches to generate the pump and probe signals. In the upper branch, a high-extinction ratio (>40 dB) electro-optic modulator (EOM), driven by a microwave signal and operating in carrier-suppression mode, generates a two-sideband probe signal. A polarization switch is used to compensate for the polarization-induced fading affecting the Brillouin gain along the fibre. The probe power launched into the fibre is set to −6 dBm to avoid unwanted spectral distortions resulting from non-local effects as reported in ref. 23. In the lower branch, a high on–off ratio (> 50 dB) pump pulse of 20 ns is generated by a semiconductor optical amplifier (SOA) and then boosted by an erbium-doped fibre amplifier (EDFA) up to 100 mW (limit imposed by modulation instability, as indicated in ref. 31). The sensing fibre is a 50-km-long standard single-mode fibre. On the receiver side, a narrowband (10 GHz) fibre Bragg grating (FBG) is inserted to select the lower-frequency probe sideband, which is detected by a 125-MHz photoreceiver. Time-domain traces are then collected using an acquisition card connected to a computer.

**Figure 2:**
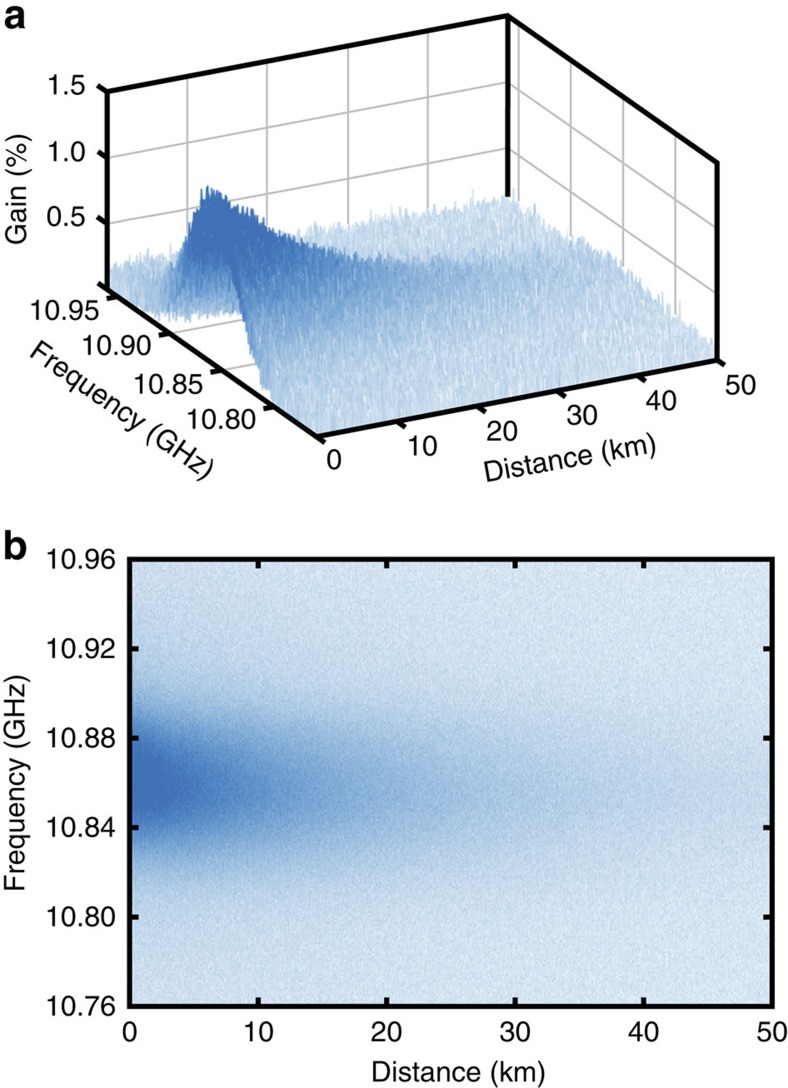
Measured BGS versus distance. (**a**) Three-dimensional map of the measured BGS as a function of distance. Measurements are obtained along a 50-km-long sensing fibre using a spatial resolution of 2 m, 4 temporal averages (2 averages per each orthogonal polarization state), a sampling interval of 0.5 m, a spectral scanning range of 200 MHz and a frequency step of 1 MHz. (**b**) Top view of the measured BGS, where pixels with darker blue tones represent position-frequency pairs with higher Brillouin gain. This image provides a visual representation of the noisy measured data and depicts the ‘image' to be enhanced by image processing.

We first tested the proposed method on measurements obtained by a standard Brillouin optical time-domain analyser^15^,^16^ (BOTDA), using the proof-of-concept experimental setup shown in Fig. 1. For a 2-m spatial resolution over a 50-km-long sensing fibre, the SNR of the implemented system turns out to be optimized using a 125-MHz high-transimpedance photodetector^57^. Although this high-transimpendance makes the system thermal-noise dominated, this leads to an optimized electrical SNR^57^. To provide a reliable demonstration of the technique proposed in this study, noisy distributed measurements are intentionally acquired using only four time-averaged traces per scanned frequency (two averaged traces for each orthogonal polarization). It is noteworthy that the acquisition procedure in Brillouin sensors makes inherently use of a 2D data structure **M**(*z*,Δ*f*) in the position *z* and frequency Δ*f* domains, from which the environmental information is retrieved by detecting spectral shifts of the peak gain frequency. Figure 2a shows the noisy 3D map of the Brillouin gain spectrum (BGS) measured along the sensing fibre for different pump–probe frequency offsets Δ*f*. In this case we use a sampling rate of 5 ns per digital point in the analogue-to-digital converter, corresponding to a sampling interval of 0.5 m per point (using for convenience the typical time-to-position conversion based on the group velocity of light in the fibre), whereas a spectral range of 200 MHz is scanned with steps of 1 MHz. The measurement process therefore results in a 2D matrix **M**(*z*,Δ*f*) of 100,000 × 200 data points containing the local BGS at each sampled fibre location. Image processing^58^,^59^ here associates each acquired position–frequency pair (*z*,Δ*f*) to a pixel (*x*,*y*) of a noisy digital image (illustrated in Fig. 2b), where *x* and *y* are the spatial coordinates of the image. This image can be represented by a two-variable function *f*(*x*,*y*) with values belonging to a 1D space, such as in a greyscale image^58^,^59^ and mapping the local Brillouin gain measured at a given position *z* and frequency offset Δ*f*. For a better visual perception of the data, a blue scale is chosen in Fig. 2, where pixels with darker tones represent position–frequency pairs having higher Brillouin gain (it is noteworthy that the scale is inverted when compared with the traditional representation of monochrome images^58^,^59^). A high level of redundancy in the signal amplitude associated to given position–frequency pairs can be found along the entire 2D data structure **M**(*z*,Δ*f*); this actually becomes evident if we consider that the same BGS (having a known spectral shape) is repeatedly measured along the fibre, being only spectrally shifted at positions where the local environmental conditions change^16^,^17^. Although the fibre attenuation can alter the BGS peak amplitude with distance, Fig. 2 shows that the matrix **M**(*z*,Δ*f*) can still be decomposed into small 2D patches containing several closely located longitudinal points having essentially the same average amplitude.

To exploit the high level of similitude and redundancy present in the 2D domain of the data, we here use two of the best-known image denoising methods as proof-of-concept, to evaluate the effectiveness of the proposed approach: the so-called non-local means (NLM)^59^,^60^,^61^,^62^,^63^ and wavelet denoising (WD)^64^,^65^,^66^,^67^ (see Methods for details). Whereas the former method operates in the spatial domain of the image, that is, making direct use of the measured data points (raw data), the later method converts the raw data into the wavelet domain (corresponding to a particular representation of the spectral domain of the image), where the components associated with noise are filtered out by wavelet shrinkage using a hard thresholding function^64^,^65^,^66^. Supplementary Fig. 1 shows the ‘denoised images' resulting after processing the raw measured data points. Although a simple visual inspection of these images indicates a clear improvement in the data quality (compared with Fig. 2b), we evaluate the effectiveness of the denoising using an objective metric by calculating the SNR of the time-domain trace obtained at the peak Brillouin gain frequency^24^. To allow a fair comparison with existing techniques, it is worth mentioning that we use here the definition of SNR calculated to be proportional to the trace amplitude, which means the ratio between the mean amplitude of the measured local response and its standard deviation^24^,^25^,^51^,^52^,^53^, contrarily to some works where the SNR is defined to be proportional to the electrical power^16^,^45^,^50^.

**Figure 3:**
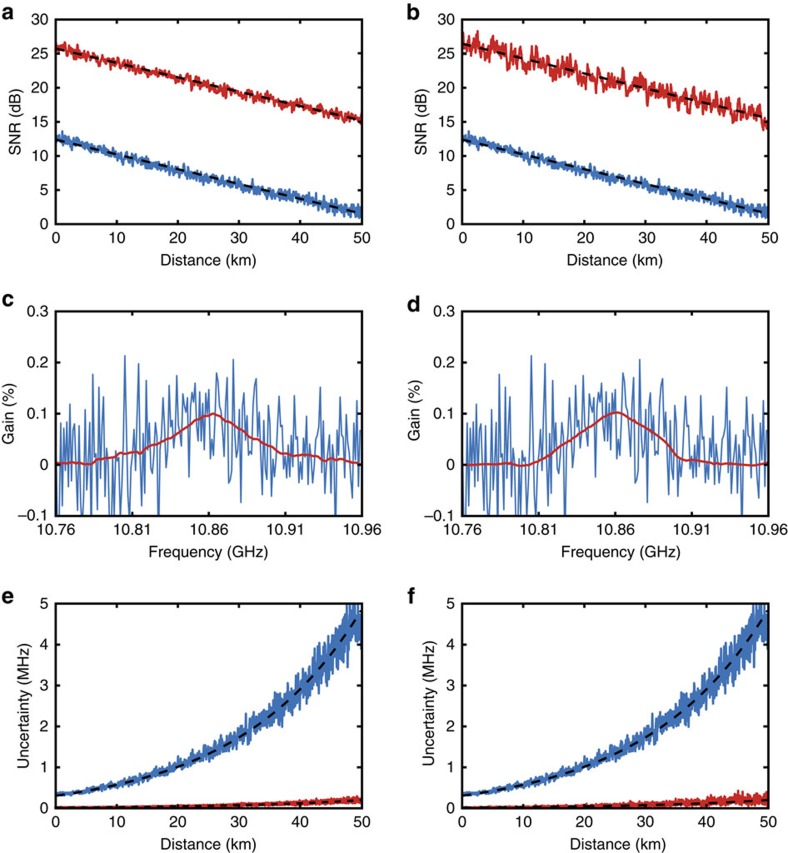
Impact of 2D image processing on BOTDA measurements. The sensing performance enhancement provided by the 2D NLM (left) and 2D WD (right) methods is evaluated by comparing the raw (blue curves) and processed (red curves) data. (**a**,**b**) SNR versus distance, showing that the raw SNR of 1.4 dB (obtained at 50 km distance with 2 m spatial resolution and 4 averaged traces) can be improved up to 15.2 and 15.6 dB with the NLM and 2D WD methods, respectively. The black dashed lines show a linear fitting (in dB scale) of the SNR curves versus distance. (**c**,**d**) BGS measured near the far fibre end, pointing out that a significant increase in the measurement contrast can be obtained by image processing. (**e**,**f**) Uncertainty on the determination of the peak gain frequency versus distance, showing that the uncertainty of 4.8 MHz obtained in the raw data at 50 km can be significantly reduced down to 0.20 and 0.19 MHz with the NLM and 2D WD methods, respectively.

**Figure 4:**
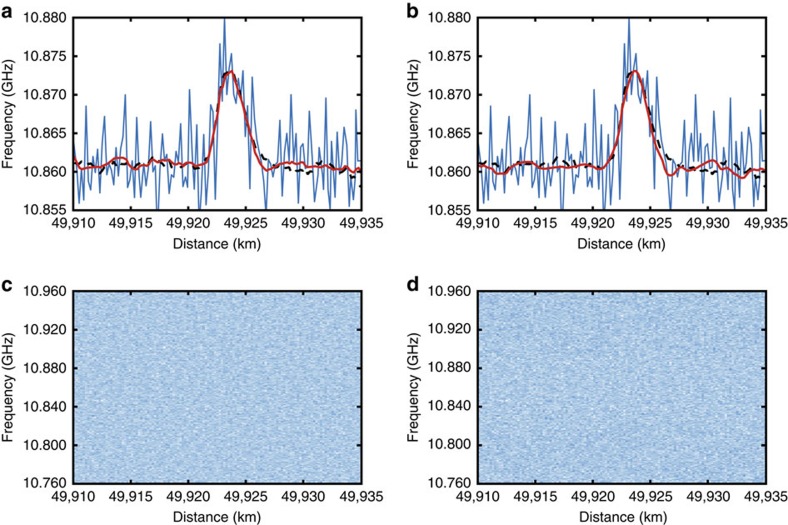
Validation of the maintained spatial resolution. Impact of the 2D image processing technique on the spatial resolution of the implemented BOTDA sensor, for the 2D NLM (left) and 2D WD (right) methods. (**a**,**b**) BFS profiles around a 2-m-long hotspot located near the far end of the sensing fibre, for the NLM and WD methods, respectively. The black dashed lines show a reference BFS profile retrieved directly from measurements (that is, with no image processing) acquired with a much larger number (4,000) of averaged traces. Results demonstrate that the applied image processing methods have only an imperceptible impact on the spatial resolution of the sensor. (**c**,**d**) Two-dimensional representation of the noise component eliminated by 2D NLM and 2D WD methods. These figures are obtained subtracting the ‘denoised images' from the ‘original image' (2D data matrix with the raw BOTDA traces), in the case of a hotspot measurement. Results show only the fibre section where the hotspot is located, indicating that the eliminated noise components do not contain any evidence of visual patterns or relevant information that could affect the proper hotspot detection.

Figure 3a,b compare the SNR of the raw noisy traces (blue lines) and the ones obtained after denoising (red lines) with the NLM and WD methods, respectively. Black dashed lines correspond to the respective linear fitting (in dB scale) of the SNR versus distance. Experimental results point out that the SNR of 1.4 dB obtained at 50 km distance on the raw data can be substantially boosted up to 15.2 and 15.6 dB by applying the NLM and WD methods, respectively. It is noteworthy that those values represent a remarkable SNR enhancement of 13.8 and 14.2 dB using each of the respective methods. Although the SNR improvement provided by these two denoising methods is fairly equivalent, we should mention that the computational complexity of the NLM is normally much larger, especially in our implementation (see Discussion section), giving a crucial advantage to the WD, unless dedicated programming and implementation are used for the NLM processing^63^. It is also important to mention that although the Brillouin frequency shift (BFS) of the sensing fibre is in this case longitudinally quite uniform, the reported SNR improvement is obtained by the NLM and WD methods using parameters that secure a spatial resolution of 2 m, as described in the Methods and demonstrated hereafter in Fig. 4.

The SNR enhancement demonstrated above has actually a massive impact on the quality of the obtained BGS. Figure 3c,d highlight the huge contrast enhancement in the BGS measurements and the considerable noise reduction provided by image processing. No relevant distortion can be observed in the BGS obtained after processing. An accurate distributed BFS profile is then obtained by fitting a quadratic curve to the BGS obtained at each fibre location^24^. The ultimate impact of image processing on the measurement quality turns evident when calculating the standard deviation of the retrieved BFS. Figure 3e,f show that the frequency uncertainty of 4.8 MHz obtained with the raw data at 50 km distance can be remarkably improved down to 0.20 and 0.19 MHz using the NLM and WD methods, respectively. These values are actually in perfect agreement with the uncertainty expected from the experimental SNR^24^ obtained after denoising.

To corroborate that the proposed technique does not actually lead to a penalizing loss of information (for example, loss of spatial resolution capabilities) and eliminates mostly the uncorrelated noise present in the measurements, it is essential to demonstrate that the applied 2D processing does not excessively blur the ‘denoised images'. For this purpose, a 2-m section of fibre at 50 km distance is heated up to 40 °C, while the rest of the fibre is kept at room temperature (27 °C). Figure 4a,b show the BFS profiles around the hotspot location obtained from the raw and denoised data for the NLM and WD methods. To accurately assess the impact of image processing on the spatial resolution, the sampling interval in this figure is reduced down to 0.2 m, while a reference BFS profile of the hotspot (black dashed line in the figures) is obtained using 4,000 averages and no processing, thus providing a reliable reference profile with comparable SNR. Results highlight the correct detection of the 2-m-long hotspot, demonstrating that the applied processing has an imperceptible impact on the spatial resolution even under low SNR conditions. We should emphasize that this verification of the spatial resolution is obtained with the same denoising parameters used to process the raw data when estimating the SNR improvement reported in Fig. 3a,b, and therefore they fully represent the spatial resolution capabilities reached by the NLM and WD techniques when enhancing the SNR by ∼14 dB. This clearly demonstrates the benefits of the proposed method when, for instance, compared with classical low-pass filtering techniques, which are highly affected by the tradeoff between noise reduction and spatial resolution. In contrast to low-pass filtering, the here-proposed technique uses a nonlinear approach, in which no explicit bandwidth notion is relevant; the amount of removed noise depends exclusively on the frequency components of the useful signal and their level of redundancy. The redundancy of information increases the amplitude of the frequency components inherent to the useful signal, while low-amplitude components are assumed to be noise and removed by the processing. This nonlinear denoising approach enables us to measure events with a sharp spatial resolution (that is, maintaining high-frequency components), while reducing significantly the noise (that is, random and low-amplitude components present in the low and/or high frequency range). Thus, fundamentally there is no discernible bandwidth change between raw and processed data, as proved by the hotspot measurements in Fig. 4a,b. More quantitatively, if we consider the spatial resolution of 2 m and the sampling interval of 0.5 m, a digital low-pass filter of maximum four points could be used (equivalent to a four-point moving average window). This leads to a 3-dB SNR improvement, which is much lower than the 14-dB improvement here demonstrated. Considering that the electrical bandwidth in the system is 125 MHz, an electrical low-pass filter of 50 MHz bandwidth could still be used to secure a spatial resolution of 2 m, but this has also minor impact on the SNR. Figure 4c,d actually confirm that the eliminated 2D component (obtained subtracting the ‘denoised image' from the original ‘noisy image') is essentially white noise, showing no specific features or patterns that could indicate the elimination of relevant information.

### Extending the concept to video processing

The principle of distributed fibre sensing assumes that the temporal evolution of the measurand changes slowly compared with the acquisition time. In the case of Brillouin and Rayleigh sensors, this leads to consecutive 2D measurements containing highly correlated information. Based on this feature, the concept proposed in this study can be extended to the 3D case^60^,^61^,^62^, in which each measurement (in the position–frequency domain) is assimilated to a frame of a video sequence. This approach exploits not only the redundancy found in the 2D domain of the measurements but also in the temporal dimension, thus leading to a very powerful tool for a better data restoration and noise removal in distributed fibre sensing. Using the same setup reported in Fig. 1, we acquire consecutive 2D data matrices **M**(*z*,Δ*f*) every ∼42 s. Although this measurement time can be still reduced due to the small averaging number (four averaged traces per scanned frequency), the response time of the equipment used in this lab demonstration eventually limited our minimum acquisition time.

Considering that the temporal evolution of the measurand might reduce the correlation existing between consecutive measurements, the effectiveness of video processing^60^,^61^,^62^ is experimentally verified under conditions in which the fibre temperature changes during the measurement process. It is important to bear in mind that if the measurands were completely static (that is, showing no temporal variations), an excellent and trivial solution to increase the SNR would be the use of a simple (linear) temporal averaging of consecutive measurements. However, owing to the dynamical nature of the environment, such a kind of averaging leads to ‘blurred images', resulting in loss of information and details, as depicted in Supplementary Fig. 2 (see description in next paragraphs). Video processing here takes into account the non-stationary characteristics of the data in the temporal domain^60^,^61^,^62^.

**Figure 5:**
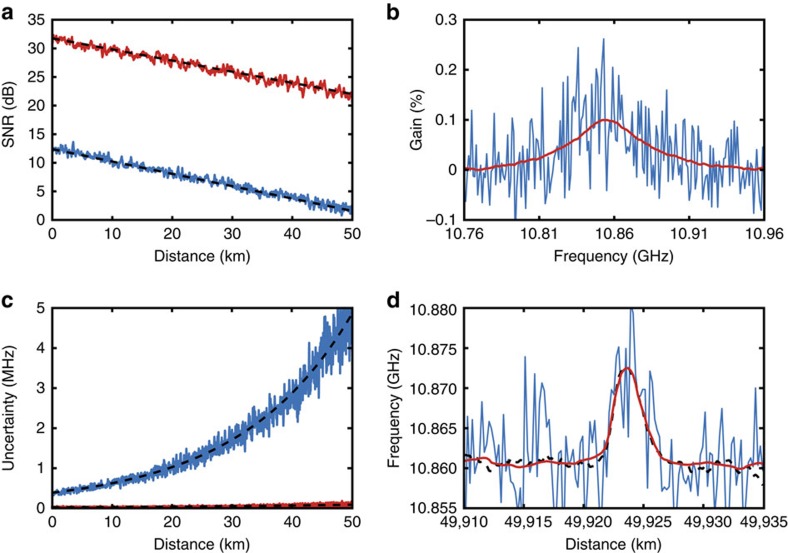
Impact of video processing on BOTDA measurements. The sensing performance enhancement provided by the 3D NLM methods is evaluated by comparing the raw (blue curves) and processed (red curves) data for different parameters. (**a**) SNR versus distance, showing that the raw SNR of 1.4 dB can be improved up to 22.1 dB when 10 consecutive measurements are used for denoising. (**b**) BGS measured near 50 km distance. (**c**) Uncertainty on the BGS peak frequency versus distance, showing that the uncertainty of 4.85 MHz obtained in the raw data at 50 km distance can be significantly reduced down to 0.055 MHz. (**d**) BFS profile around a 2-m-long hotspot located near the far end of the sensing fibre. Negligible impact on the spatial resolution of the sensor can be observed. The black dashed line in **d** shows a reference BFS profile retrieved directly from a measurement obtained with 4,000 averaged traces.

Measurements are processed using a 3D NLM algorithm^60^,^61^,^62^ (see Methods). As in the 2D case, the 3D spatio-temporal NLM method uses the self-similarity of an image, but the search for repeated patterns is extended to several consecutive frames^60^,^61^,^62^. Figure 5a shows the SNR of the trace measured at the peak gain frequency for the raw (blue curve) and denoised (red curve) data using the 3D NLM method^60^,^61^,^62^ based on ten consecutive measurements (frames). The figure indicates that the raw SNR of 1.4 dB obtained at 50 km distance can be increased up to 22.1 dB using 3D NLM processing. This is >6.5 dB improvement with respect to the 2D image processing reported above and corresponds to a remarkable absolute SNR enhancement of 20.7 dB. The benefits provided by this SNR enhancement can be clearly observed in Fig. 5b, which shows the BGS measured at 50 km distance obtained from the raw and denoised data. Figure 5c highlights that the BFS uncertainty of 4.85 MHz, obtained from the raw data, can be significantly reduced down to 0.055 MHz with this 3D NLM processing, being in good agreement with the attained SNR enhancement^24^.

Supplementary Movie 1 shows the BFS profile along the last metres of fibre measured when the temperature of a 2-m section changes from 10 °C up to 40 °C (the rest of the fibre is at 27 °C). As also shown in Fig. 5d and Supplementary Fig. 2, results demonstrate that the retrieved BFS profiles present no observable distortion of the hotspot, while negligible delay is observed when compared with the temperature evolution retrieved from the raw measurements. This represents a key advantage of video processing when compared, for example, with linear temporal averaging, which generally leads to delays in the temporal evolution of the measurand, as demonstrated in Supplementary Fig. 2.

### Denoising 1D data using 2D image processing

**Figure 6:**
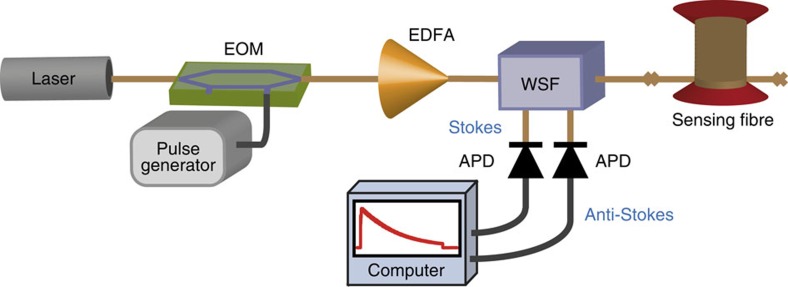
Experimental setup of a basic Raman-distributed sensor. The system operates using a conventional distributed-feedback laser operating at 1,552 nm, followed by an electro-optic modulator (EOM) to generate pulses of 10 ns and an erbium-doped fibre amplifier (EDFA) to boost the pulse power. Optical pulses of about 4W peak power are launched into the sensing fibre through a wavelength-selective filter (WSF), which also separates the spontaneous Stokes and anti-Stokes Raman components backscattered from the sensing fibre into two branches. These two spectral components are sent into two parallel 50 MHz avalanche photodetectors (APD), followed by an acquisition system connected to a computer. The sensing fibre corresponds to a 50/125-μm graded-index multimode fibre 9 km long. As a result of intermodal dispersion, the spatial resolution at the end of the sensing fibre is ∼2 m.

In sensors measuring only 1D data, such as Raman-distributed temperature sensors, a 2D image can be formed considering time as a second dimension when stacking successive sequential measurements. To validate the proposed method in this case, we use a generic Raman-distributed sensor scheme^12^,^13^,^14^, as depicted in Fig. 6. Using a 9-km-long sensing fibre, a spatial resolution of 2 m and a sampling interval of 0.5 m, we measure 1D time-domain traces of the spontaneous Raman anti-Stokes and Stokes backscattered components using 4,096 averages, within a measurement time of ∼25 s. Traces are consecutively measured, while the temperature of ∼10 m of fibre (at the end of the sensing range) is changed from room temperature (∼25 °C) up to 40 °C.

**Figure 7:**
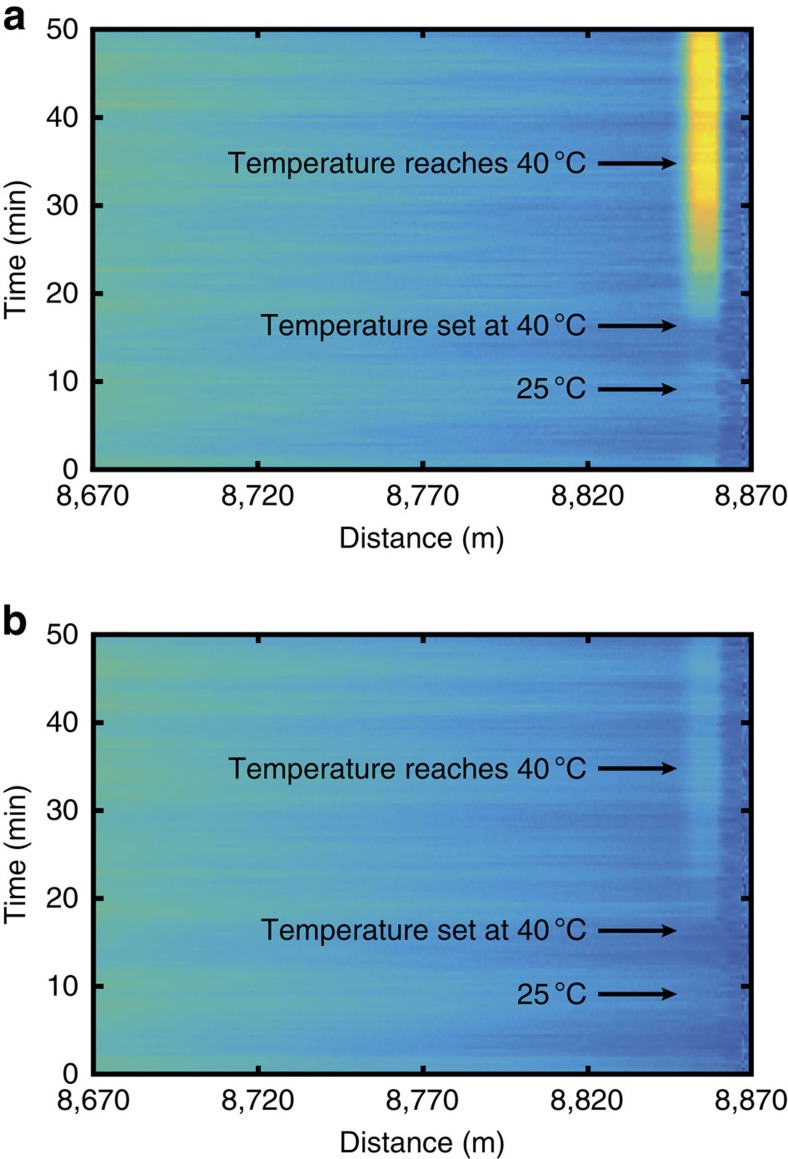
Two-dimensional images formed from stacked 1D Raman time-domain traces. The images show excerpts from the time-domain traces of the spontaneous Raman (**a**) anti-Stokes and (**b**) Stokes components, measured using a 9-km-long sensing fibre. During the measurement process, the temperature of 10 m of fibre has been increased from room temperature (25 °C) up to 40 °C. For the sake of clarity, only the last 200 m are shown in the figure.

As suggested, we form two 2D images (one for the Stokes and another for the anti-Stokes signal) stacking together consecutive time-domain traces. Figure 7a,b provide 2D image representations of the evolution of consecutive Raman anti-Stokes and Stokes traces within the last 200 m of fibre. The temperature evolution affecting the anti-Stokes trace amplitude around 8.86 km is evident in Fig. 7a. Although the entire set of data contained in those images could be used for denoising, a sliding temporal window of 21 consecutive measurements is chosen here. Thanks to this restricted temporal window, the processing time is highly reduced without significantly compromising the denoising capabilities of the method^63^. Thus, to remove noise from a ‘current' measurement, matrices **M**_**aS**_(*z*,*t*) and **M**_**S**_(*z*,*t*) of 18,000 *×* 21 points (including the raw measurements of the ‘current' and previous 20 anti-Stokes and Stokes traces) are processed by the 2D NLM method. This procedure is continuously repeated for each new measured trace and independently for the Stokes and anti-Stokes signals. After applying image denoising to the raw measurements, the ratio anti-Stokes to Stokes is calculated at each fibre location, and by following a standard calibration procedure the actual distributed temperature profile is finally obtained^12^,^13^.

**Figure 8:**
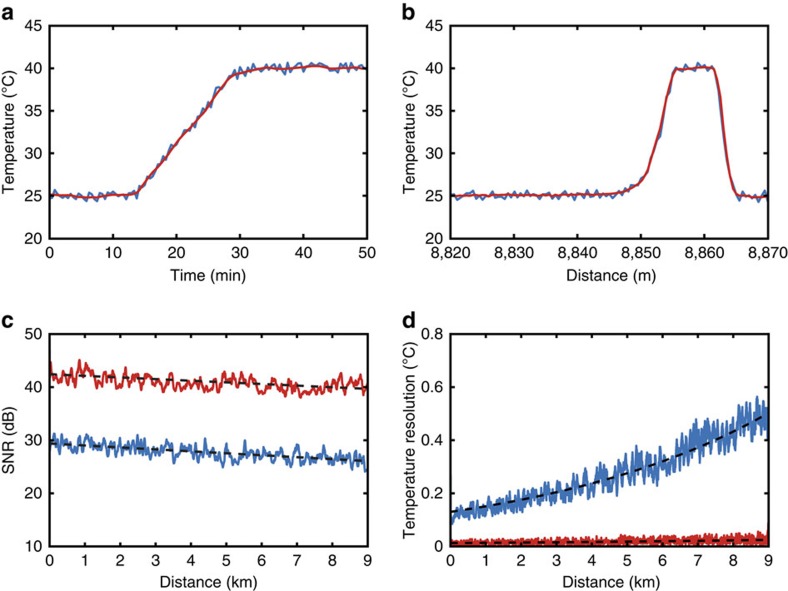
Impact of 2D image processing on 1D Raman traces. The performance of the sensor and the impact of 2D image denoising is evaluated while the temperature of 10 m at the end of the sensing fibre is slowly increased in time. Results obtained from the raw data (blue) are compared with the ones obtained from the denoised data using the 2D NLM method (red curve), in which a moving window of 21 consecutive traces is considered. (**a**) Temporal evolution of the measured temperature at the hotspot location; this result shows that image processing induces no delay or distortion in the measured hotspot temperature. (**b**) Distributed temperature profile near the hotspot location, demonstrating that the denoising process produces no perceptible loss of spatial resolution. (**c**) SNR versus distance, validating an SNR enhancement of 13.6 dB at the end of the sensing range. (**d**) Temperature resolution versus distance, showing that the use of 2D NLM method can improve the temperature uncertainty from 0.5 °C down to 0.022 °C.

Figure 8a shows the temporal evolution of the measured temperature within the hotspot section, whereas Fig. 8b shows the measured temperature profile around the hotspot after the fibre temperature reaches a stable temperature of 40 °C. Results demonstrate that no loss of spatial resolution and no distortion or delay in the temperature evolution are induced by the processing. Figure 8c shows that the SNR of 26.2 dB obtained at 8.86 km distance with the raw data can be significantly improved up to 39.8 dB after 2D NLM denoising. This corresponds to an SNR enhancement of 13.6 dB, which leads to a temperature resolution improvement from 0.5 °C (obtained from raw data at the fibre end) down to 0.022 °C after NLM processing, as shown in Fig. 8d.

## Discussion

In this study, we have proposed and demonstrated the use of image/video denoising techniques^58^,^59^,^60^,^61^,^62^,^63^,^64^,^65^,^66^,^67^ as an efficient approach to enhance the SNR of distributed fibre sensors. To the best of our knowledge, this is the first time that the high levels of correlation and redundancy contained in the multidimensional domain of the measurements obtained by distributed fibre sensors are exploited for performance improvement. Compared with state-of-the-art methods, the multidimensional processing approach here proposed turns much more efficient than applying known (1D) denoising algorithms^44^,^45^,^46^,^47^,^48^,^49^,^50^,^51^ simply replicated in the different dimensions of interest. For instance, the independent use of 1D processing to denoise time-domain traces and then applied to the processed data in frequency domain leads to denoised data points that do not benefit from the similitude and redundancy that can only be found in a 2D or 3D data structure containing the entire measured information. For this reason, the method here proposed offers exceptional denoising capabilities when compared with state-of-the-art techniques^33^,^34^,^35^,^36^,^37^,^38^,^39^,^40^,^41^,^42^,^43^,^44^,^45^,^46^,^47^,^48^,^49^,^50^,^51^,^52^,^53^,^54^, enabling a remarkable and unprecedented SNR enhancement, boosting the sensor performance^24^ up to about two orders of magnitude with no loss of relevant information at minor added cost. This translates, for instance, into an unmatched 100-fold improvement in the measurand accuracy of conventional distributed sensors. Furthermore, this multidimensional processing can be freely implemented on top of existing sophisticated methods for performance improvement^33^,^34^,^35^,^36^,^37^,^38^,^39^,^40^,^41^,^42^,^43^ such as optical pulse coding and/or distributed Raman amplification, thus resulting in a fully additional SNR enhancement.

Although the Brillouin system used in this study as a proof-of-concept is thermal-noise dominated, it is worth mentioning that the efficiency of the proposed technique would be similar if shot noise, spontaneous-signal beat noise (when using an erbium-doped fibre amplifier in the receiver) or relative intensity noise (RIN) had an impact on the measurements^57^. In fact, the largest contribution to noise in those cases is given basically by the continuous-wave probe power reaching the receiver^25^,^57^, whereas the response of the BOTDA trace only corresponds to a very small fraction of this continuous-wave level (typically 1% for 1 m spatial resolution), having a negligible impact on the signal noise. Such sources of noise can therefore as well be considered additive and uniform along the fibre^57^. When RIN dominates, traces can be affected by noisy patterns that could partially impact on the gained SNR of the non-local means and WD methods used in this study; however, we should mention that there exists a vast variety of image processing algorithms and some of them could be more efficient for removing RIN. Further research on this subject is still necessary and goes beyond the scope of this study. If Brillouin time-domain traces are affected by stationary patterns, for instance, when polarization fading are imperfectly compensated, then image processing will consider those patterns as real signal, leading to a non-uniform noise removal along the fibre. However, as fading are proportional to the Brillouin gain, they would eventually affect the noise removal along the first kilometres of fibre, where the SNR is still high, and therefore under normal operation conditions there should be no real detrimental effects at long distances (low SNR region dominated by white noise) where the benefit from image denoising is more crucial. Furthermore, if slow-varying temporal instabilities affect the sensor operation, for instance, due to thermal fluctuations, the effectiveness of the denoising might also be reduced when the temporal dimension is considered in the processing. This is because slow-varying fluctuations and instabilities could be interpreted by image/video processing as a correlated signal containing relevant (that is, non-random) information, thus imposing an ultimate limit to the estimated SNR improvement.

Although the concept proposed in this study has only been demonstrated for BOTDA and Raman sensors, it is envisaged that image/video processing can also be applied to improve SNR of other sensors, such as Brillouin optical-time domain reflectometers^21^, as well as schemes based on frequency domain^18^ and correlation domain^19^,^20^. Furthermore, the concept can also be extended to Rayleigh-based sensors^7^,^8^,^9^,^10^,^11^, such as phase-sensitive optical time-domain or frequency-domain reflectometers for quasi-static strain and temperature sensing, where image/video processing can be applied to the 2D data matrix containing the calculated correlation spectrum versus distance^9^,^10^,^11^. Although correlation noise could dominate the correlation spectrum in that case, some dedicated image/video processing algorithm could still provide an important enhancement in the contrast of the main correlation peak. This certainly requires a further study and goes beyond the main scope of this study. In addition, the approach followed for denoising 1D traces can also be applied to standard reflectometry measurements^5^,^6^, including even more sophisticated methods for fibre characterization.

An interesting aspect that should be highlighted is that the 3D approach here proposed has proved to be very efficient when dealing with the dynamical character of the measurements. This is because video sequences are also inherently non-stationary^60^,^61^,^62^ and therefore conventional video processing can easily deal with the motion of pixels among different frames. This, if properly tackled and implemented for instance in a dedicated graphic processing unit, could have interesting applications in dynamic distributed sensing^22^. Nevertheless, we should clarify that video denoising typically follow two different approaches: (i) the use of methods with no motion compensation^60^,^61^,^62^ and (ii) the use of methods with explicit motion compensation^68^,^69^,^70^. Whereas the 3D NLM^60^,^61^,^62^ used here as a proof-of-concept belongs to the first group, there exist also several movie denoising techniques belonging to the second category, such as the 3D WD^68^,^69^,^70^. Further investigation can be still carried out to verify the effectiveness of methods based on explicit motion compensation^68^,^69^,^70^. However, we should mention that the concept of calculating the motion of pixels, that is, the trajectory of pixels through consecutive frames, is already inherently incorporated in the 3D NLM method^60^,^61^ and therefore already demonstrated in this study.

It is also worth mentioning that the experimental results shown in this study have been obtained by simple image/video denoising implementations, where parameters have been selected by following standard recommendations and fine empirical adjustments to maximize the noise removal avoiding any loss of relevant information. However, more dedicated strategies for adjusting parameters could be still developed and further investigated. For instance, the possibility for auto-tuning parameters and adaptive window sizes to avoid oversmoothing fast spatial and temporal changes of the measurand could also be investigated.

The processing time of the method could also be further investigated and improved. No special hardware has been here used to optimize the processing time. Considering the large number of acquired points in the demonstration sensors, the implementation of a standard NLM algorithm turns out to be computationally very demanding. Using a conventional computer with a 3.5-GHz processor and 8 GB RAM, the 2D NLM processing time of each matrix of 18,000 × 21 points in the implemented Raman sensor turns out to be about 1 s. On the other hand, denoising the 2D data matrix of 100,000 × 200 points in the BOTDA sensor takes about 30 s: this is about one order of magnitude larger than the time required by the WD, which requires <1 s for processing the same data. Furthermore, the 3D NLM denoising of the same matrix size, but considering ten consecutive frames, is about 4 min. This time can however be highly reduced following specific strategies^62^,^63^: for example, using parallel computation to distribute the operations on several processors. In fact, the NLM method is intrinsically well suited for parallelization and multithreading^63^, making this kind of approach very efficient and straightforward. Much further improvement can also be obtained if dedicated algorithms and programming are used to implement image/video denoising in a dedicated graphic processing unit installed in a sensor system. This shows that we are just at the early stages of research, only unveiling a part of the promising potential of these techniques.

Although in this study we demonstrate only the effectiveness of image denoising methods, other techniques for image enhancement^58^ (different from denoising) can also be employed to increase the measurement quality of distributed fibre sensors in some particular conditions or to spot particular events in a massive data flow. This can be obtained using dedicated algorithms^58^, for instance, to sharpen image details, increase the dynamic range of particular features, restore blurring effects, enhance contrast and edges, and several other approaches. Many of those methods actually offer the possibility to recognize objects or patterns, which could be very helpful for the detection of special features that could be present in the measurand. Blur-removing strategies could also be investigated to sharpen the detection of small events, comparable to or potentially shorter than the spatial resolution of the sensor.

## Methods

### Non-local means

This technique^59^,^60^,^61^,^62^,^63^ proposes an original paradigm in noise reduction by taking advantage of the high degree of redundancy contained in the 2D or 3D data measured by a distributed fibre sensor. The method is based on the use of sliding neighbourhoods or 2D patches, which correspond to sets of all pixels *j*=(*x*,*y*) that surround a certain pixel at 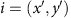 within a window of a predefined size. The similarity between two pixels *i* and *j* is performed by comparing, not only the values 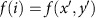 and *f*(*j*)=*f*(*x*,*y*) assigned to the pixels but the entire 2D patches or neighbourhoods surrounding the pixels of interest^59^,^60^. For this, a so-called similarity neighbourhood **η**_*i*_ surrounding the pixel *i* is defined and then compared with all other similarity neighbourhoods **η**_*j*_ of the same size existing in the entire 2D matrix containing the data provided by the sensor.

The degree of similitude and redundancy in the data is evaluated in a patch-by-patch basis by calculating the Euclidean distance^59^,^60^ ||*f*(**η**_*i*_)-*f*(**η**_*j*_)|| between all values *f*(**η**_*i*_) and *f***(η**_*j*_) within neighbourhoods **η**_*i*_ and **η**_*j*_. It turns out that the Euclidean distance is small when a high level of similitude exist between both compared windows **η**_*i*_ and **η**_*j*_, and therefore the highly similar values *f*(*i*) and *f*(*j*) associated to both pixels *i* and *j* can be averaged to reduce noise^59^,^60^. More specifically, to eliminate noise from a pixel *i* in the 2D data matrix, the value *f*(*i*) associated to such a pixel is processed by the NLM method calculating the following weighted average^59^,^60^:


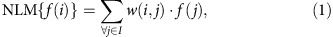


where *I* is the entire domain of the image, *f*(*j*) corresponds to the value of the image associated to the pixel *j* and *w*(*i*,*j*) are the weighting factors calculated as^59^,^60^


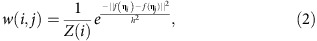


where *h* is a smoothing control parameter and *Z*(*i*) is a normalization factor defined so that the sum of all values of *w*(*i*,*j*), for a given pixel *i*, results to be equal to one. It should be noted that the weighting factors *w*(*i*,*j*) are independent of the geometry and only depend on the similarity of the data around pixels *i* and *j*. This feature characterizes the method as non-local, as pixels *j* whose surroundings are similar to the pixel *i* are associated to a higher weight *w*(*i*,*j*), regardless of the relative (spatial) distance between the two pixels.

A strict condition for the processing is that the similarity window size must be comparable to the smallest details in the image^59^,^60^, which in the context of distributed fibre sensing is associated to the spatial resolution capabilities of the sensor. Considering that all implemented and analysed systems in this study (Brillouin and Raman sensors) have a spatial resolution of 2 m and a sampling interval of 0.5 m, the similarity window has been chosen of size 3 *×* 3. This size, corresponding to three longitudinal data points (that is, 1.5 m long), is smaller than the spatial resolution of the system, ensuring that the processing does not have any detrimental impact on the real spatial resolution of the sensor.

On the other hand, the parameter *h* controls the level of blurring of the method and its optimum value depends on the noise level of the data. Following the recommendation given in ref. 59, *h* has been set to ten times the noise standard deviation *σ*. In the Brillouin gain data shown in Fig. 2, the noise standard deviation is *σ=*7.2 × 10^−4^; thus *h* has been set to 7.2 × 10^−3^. In the case of the Raman sensor implemented in this work, the noise standard deviation of data in Fig. 7 is evaluated to be *σ=*6.9 × 10^−4^ and therefore *h* has been set to 6.9 × 10^−3^.

Furthermore, we should consider the large amount of data obtained by the implemented sensors. For instance, in the case of the implemented BOTDA sensor 20 M points (that is, 20 Mpix) are acquired to cover 50 km of sensing fibre, sampled every 0.5 m and scanning 200 frequencies, so that the calculation of equations (1) and (2) turns out to be extremely demanding. To reduce the processing time, the strategy proposed in ref. 59 is applied, in which a spatially constrained search window is defined. This window is larger than the similarity window, but smaller than the entire 2D matrix (image) so that the search for repeated patterns is only restricted to the area covered by this search window. In practice, using a similarity window of 3 *×* 3 and *h*=7.2 × 10^−3^, it is empirically observed that a satisfactory noise removal is obtained at a reasonable computational cost when defining a search window of 13 *×* 13. Increasing this search area has only led to a marginal SNR improvement, at the cost of a substantial increase in the processing time.

As in the 2D case, the 3D spatio-temporal NLM method^60^,^61^,^62^ uses the self-similarity of an image to reduce the noise of a pixel 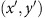 by averaging weighed image sections at coordinates (*x*,*y*) that have high level of similarity; however, in this case the search for repeated patterns is also extended to several consecutive frames^60^,^61^,^62^. As the spatial features of the acquired BOTDA data are maintained, the same parameters as in the 2D case are used for video denoising, but with the difference that the search for repeated patterns is extended to ten consecutive measurements.

Finally, in the case of processing 1D data provided by the implemented Raman distributed sensor, two 2D images (one for the Stokes and another for the anti-Stokes component) are formed by stacking together consecutive time-domain traces. A sliding search window of size 23 *×* 23 is used for 2D NLM denoising.

### Wavelet denoising

The method is based on the 2D discrete wavelet transform (DWT) and a wavelet shrinkage strategy^64^,^65^,^66^, and has been applied only to BOTDA measurements as a proof-of-concept. To eliminate noise using a 2D WD approach, data provided by the BOTDA sensor are decomposed using the Mallat algorithm^67^ into versions of sub-images containing different levels of details. After testing many mother wavelets, the wavelet sym7 has been chosen because of the better denoising capabilities obtained in this case, while the number of levels of decomposition has been set to 5. By comparing the wavelet coefficients obtained in the 2D DWT with a predefined threshold, wavelet shrinkage is then applied to the wavelet coefficients using a hard thresholding strategy^66^. This means that all wavelet coefficients having an amplitude below a given threshold are associated to noise and set to zero, whereas high-amplitude coefficients are associated to useful information provided by the sensor. Following the recommendation given in ref. 59, in this case the threshold level has been set to three times the noise standard deviation from which a small adjustment has been performed to optimize the amount of removed noise. Thus, considering that the noise standard deviation is *σ*=7.2 × 10^−4^ for the BOTDA measurements, the threshold value is set to 2.6 × 10^−3^ (≈3.6*σ*). The output 2D data matrix (considered as the ‘output image') is reconstructed from the result of this thresholding stage, using an inverse 2D DWT procedure, which converts the data back to the spatial domain of the image.

## Additional information

**How to cite this article:** Soto, M. A. *et al.* Intensifying the response of distributed optical fibre sensors using 2D and 3D image restoration. *Nat. Commun.* 7:10870 doi: 10.1038/ncomms10870 (2016).

## Supplementary Material

Supplementary Information Supplementary Figures 1-2.

Supplementary Movie 1 Hot-spot detection in a 50 km-long BOTDA sensor. Brillouin frequency shift retrieved from the raw (blue curve) and denoised (red curve) data, when 2 m of fibre at the end of a 50 km sensing range is heated from 10°C up to 40°C during the measurement process. Measurements are obtained with a spatial resolution of 2 m and 4 averages, with a measurement time (interval between frames) of 42 s. The 3D nonlocal means method is used considering 10 consecutive (‘current' and 9 preceding) frames for denoising.
