# Supplementary material for: Intensifying the response of distributed optical fibre sensors using 2D and 3D image restoration
Source: Nat Commun. 2016 Mar 1;7:10870. doi: 10.1038/ncomms10870 (PMC4773526; doi:10.1038/ncomms10870)
Supplement: Supplementary Information — Supplementary Figures 1-2. [file ncomms10870-s1.pdf]

Supplementary Figures

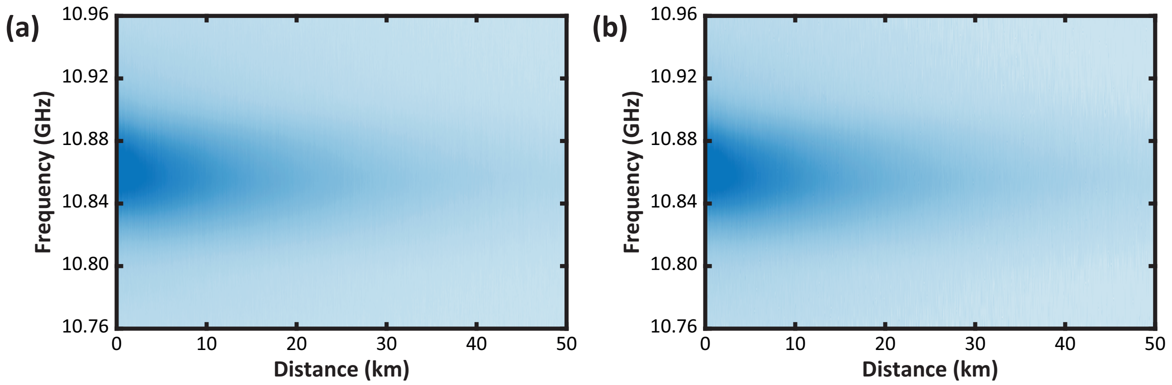

Supplementary Figure 1. Images obtained after two-dimensional denoising. The images provide a 2D representation of the denoised BOTDA data using **(a)** 2D nonlocal means and **(b)** 2D wavelet denoising methods. The parameters used for the nonlocal means method are: similarity window: $3x3$, search window $13x13$, and filtering parameter h equal to $7.2 \cdot 10^{-3}$. In the case of the wavelet denoising a hard thresholding strategy is used for the shrinkage of the wavelet coefficients (threshold equal to $2.6 \cdot 10^{-3}$) together with 5 levels of decomposition. Data contain 200 frequencies scanned every 1 MHz and 100’000 longitudinal points to cover 50 km of sensing fibre sampled every 0.5 m.

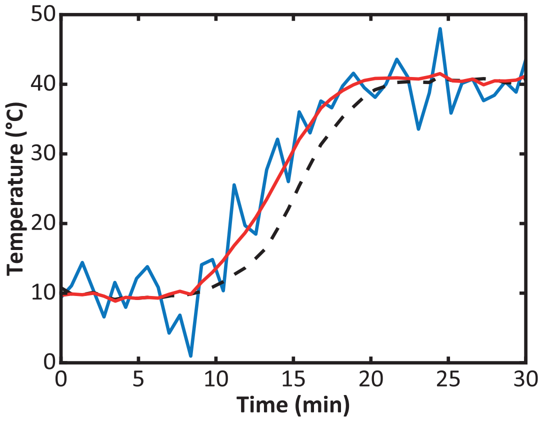

Supplementary Figure 2. Impact of video processing in the measured temperature evolution. The figure shows the temperature retrieved by the implemented BOTDA sensor in a fibre section where the fibre temperature is gradually increased during the measurement process. The temperature retrieved from the raw data (blue line) is compared with the one obtained from the denoised data using the 3D NLM method (red line), where a moving window of 10 consecutive frames is used for 3D processing. For comparative purposes, the figure also shows the temperature retrieved when using a simple linear moving window averaging of 10 consecutive measurements (black dashed line). Results highlight that, unlike the linear averaging strategy, the video processing approach induces a minimum or even negligible delay in the temperature evolution, being an essential asset of the here proposed method.
